# A growing pandemic: A review of *Nosema* parasites in globally distributed domesticated and native bees

**DOI:** 10.1371/journal.ppat.1008580

**Published:** 2020-06-18

**Authors:** Arthur C. Grupe, C. Alisha Quandt

**Affiliations:** Ecology and Evolutionary Biology, University of Colorado, Boulder, Colorado, United States of America; Vallabhbhai Patel Chest Institute, INDIA

## *Nosema* infection in bees

Domesticated and native bees face a variety of deadly threats that cause mortality and reduced fecundity and thus, by extension, endanger agriculture and native plant communities that rely on bees for pollination. Biotic factors negatively impacting bees include: viruses, nematodes, mites, bacteria, and fungi. Additionally, abiotic threats include the destruction of nesting and floral resources from anthropogenic sources as well as a plethora of negative factors from climate change. While a substantial amount of research has been done investigating the causes of colony collapse disorder in the European honey bee, *Apis mellifera*, there is growing evidence over the past two decades that another pandemic of bees, both domesticated and native, is growing. This pandemic is the result of the spread of fungal pathogens in the genus *Nosema*.

*Nosema* species belong to Microsporidia, which are all unicellular, obligate symbionts of animals, and gregarines. Although long thought to be protists, Microsporidia are now recognized as a highly reduced lineage of fungi [1]. Tokarev and colleagues [2] recently placed *Nosema* species that infect bees (Anthophila, Hymenoptera) within a new genus, *Vairimorpha*, but for the sake of consistency with the existing literature this Review article will refer to them simply as *Nosema*. Specifically, *Nosema* carry out their life cycle by infecting the cells in the midgut of bees. Once a spore is ingested by a bee and reaches the midgut, it will germinate. It then injects its contents into the host cell where it consumes the cell contents via phagocytosis until it eventually lays down spore walls before rupturing the host cell to release the spores [3]. These spores can then infect other cells in the digestive tract or be passed out of the host in excrement, thereby contaminating floral resources, collected pollen, and the nesting environment. Other bees are then susceptible to ingest spores in the nest via fecal–oral transmission, or if excreted at a floral resource, the fungus can infect any susceptible hosts that come into contact with that flower [4,5]. Due to the extent of bee foraging ranges, this process not only increases the local pathogen load but also serves to disperse *Nosema* to new habitats and novel hosts. In addition to the natural transmission of these pathogens, commercial products such as honey, bee pollen, and royal jelly can be contaminated and potentially disperse these pathogens [6].

The most common symptoms of *Nosema* infection are dysentery and microscopic lesions within the gut and Malpighian tubules. This leads to host frailty, lethargy, and loss of workers in eusocial bees that reduces foraging ability for the colony through mortality, reduced homing ability, shorter foraging flights, and inefficient foraging behavior [5,7]. *Nosema bombi* infections also reduce the fecundity of the colony through detrimental physical effects to the reproductive organs in male bumblebees, increased mortality of workers, and negatively impacting the ability of next season’s queens to found new colonies under laboratory conditions [8]. While there have been studies to observe the detrimental effects of *Nosema* infections in both *Bombus* and *Apis* species, as reviewed in Brown [9] and Martin-Hernandez and colleagues [10], almost nothing is known about the impact on native, solitary bees.

While microscopic detection of *Nosema* infections is possible, determining which species is causing the infection can be difficult. *N*. *bombi* can be morphologically differentiated from *N*. *apis* and *N*. *ceranae*, but distinguishing between these two is impossible without molecular techniques. Typically, identifying which pathogen or pathogens may be causing an infection requires specialized molecular primers for the small subunit of the rDNA cassette [11]. Through the use of these molecular primers, other species of *Nosema* have been detected in bees: *Nosema neumanni* in commercial honeybee colonies in Uganda, *Nosema cf*. *thomsoni* in *Andrena vaga* in Belgium, *Nosema thomsoni*, and *Nosema portugal* in commercial *Bombus* species in Chile and Argentina [12,13,14], although the detrimental effects of these pathogens are unclear and require further study.

## Changing distributions

Historically, *N*. *apis* and *N*. *ceranae* were found in distinct geographic locations: Europe and North America for *N*. *apis*, and South East Asia for *N*. *ceranae* [15]. With the increasing export of commercial hives from Europe, *N*. *apis* followed. For many decades, *N*. *apis* was the dominant strain infecting colonies. While it causes dysentery in *A*. *mellifera*, the seasonality of the infection cycle was such that it would not cause total devastation of the hive. Research over the past few decades, since *N*. *ceranae* was first described, has shown a dramatic increase in its contribution to the total number of *Nosema* infections in *A*. *mellifera* [16,17]. Studies have shown that *N*. *ceranae* has been replacing *N*. *apis* throughout the range of *A*. *mellifera* [18,7]. Not only has *N*. *ceranae* replaced *N*. *apis* as the main *Nosema* pathogen in *A*. *mellifera*, the lack of seasonality of *N*. *ceranae* infections has led to year-round infection cycles that are ultimately more damaging to *A*. *mellifera* hives [7]. In addition to the changing distribution of these pathogens, genomic studies have revealed that isolates from geographically distinct countries have a very high level of genetic diversity and are potentially polyploid, and local populations within its native range have a unique set of single nucleotide polymorphisms that indicate evolutionary adaption within the native range [19,20].

## Sampling of native bees

**Fig 1:**
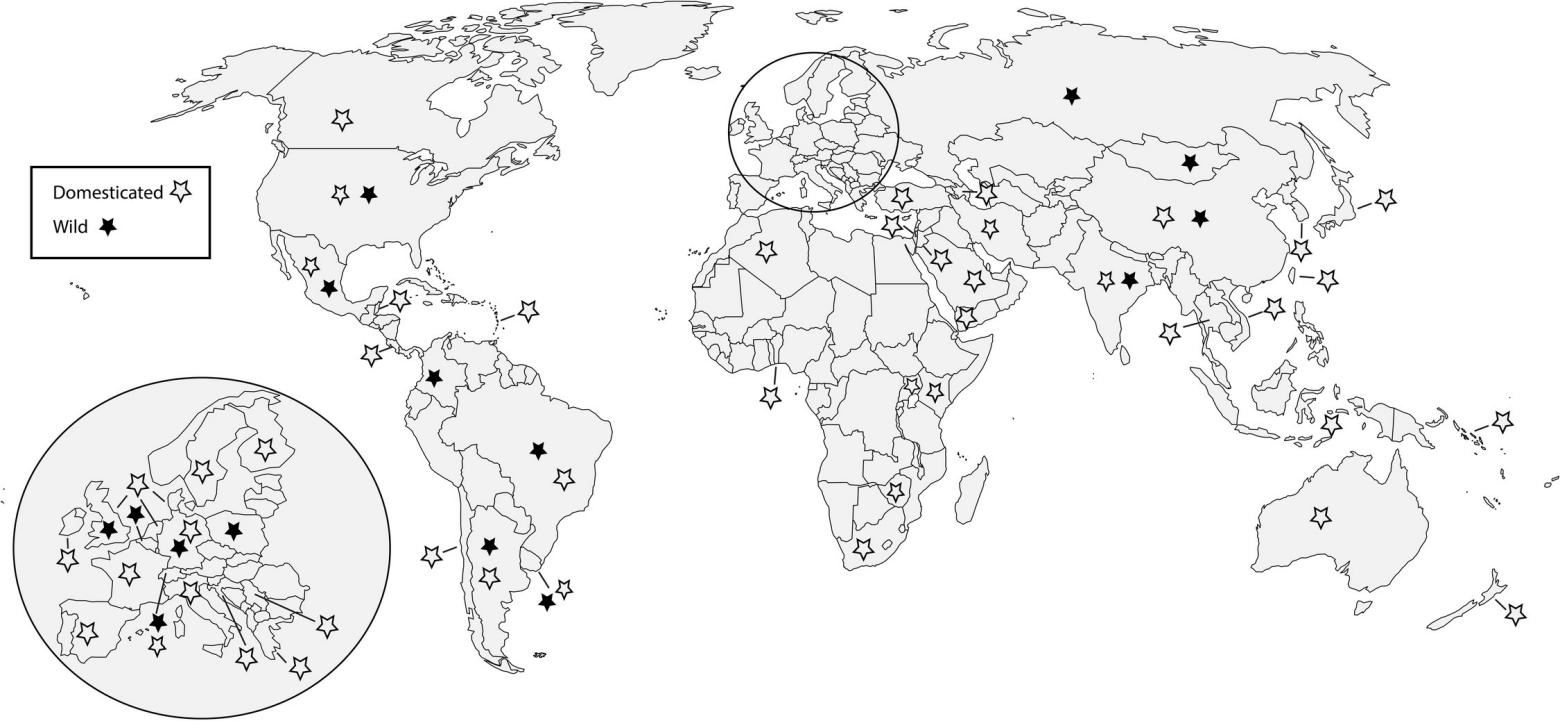
Worldwide distribution of *Nosema* species infecting bees. Distribution of *Nosema* species infecting domesticated, wild, or both bees from environmental survey studies (15,16,18, 21–24, 28–72).

While much of the work documenting the prevalence and distribution of *Nosema* species in bees of commercial interest has been done, some researchers have investigated the distribution of *Nosema* infections in native bees (Fig 1, Table 1, and S1 Table). Given the economic importance of domesticated bees to agriculture, this imbalance is understandable. However, when the ecosystem service of pollination is viewed in the wider lens of native plant communities, and the consequences of diminished pollination on community fitness, the distribution and impacts of *Nosema* species in native bees becomes a significant concern. Several studies have recognized this threat and investigated the distribution of *Nosema* species in native bees [Fig 1, 21,22,23,24]. The decline of pollination services to native plants is of concern not only in ecosystem maintenance but also conservation and restoration efforts. Furthermore, additional research is needed to determine both the pathology and distribution of infections in native bees. Through studying native bees and the distribution of *Nosema* infections in them, we can better understand the long-term consequences to native bees and the plant communities reliant on them.

**Table 1:** Host genera of bees that have a species with a documented infection of a species by *Nosema*.

Host genera	*Nosema apis*	*Nosema bombi*	*Nosema ceranae*
***Apis*** ^**D,N**^	X^[^^16^^,^^18^^,^^28^^,^^29^^,^^30^^,^^31^^,^^32^^,^^33^^,^^34^^,^ ^35^^,^^36^^,^^37^^,^^38^^,^^39^^,^^40^^,^^41^^]^ (5)	X^[^^43^^]^ (>1)	X^[^^4^^,^^13^^,^^16^^,^^18^^,^^29^^,^^30^^,^^31^^,^^32^^,^^33^^,^^35^^,^^36^^,^^37^^,^^40^^,^^41^^,^^43^^,^^53^^,^^54^^,^^55^^,^ ^56^^,^^57^^,^^58^^,^^59^^,^^60^^,^^61^^,^^62^^,^^63^^,^^64^^,^^65^^,^^66^^,^^67^^]^ (>10)
***Bombus*** ^**D,N**^	X^[^^42^^]^ (1)	X ^[^^23^^,^ ^24^^,^^44^^,^^45^^,^^46^^,^^47^^,^^48^^,^^49^^,^^50^^,^^51^^,^^52^^]^ (>53)	X^[^^4^^,^^21^^,^^22^^,^^42^^,^^47^^,^^68^^,^^69^^,^^70^^,^^71^^]^ (21)
***Andrena*** ^**N**^			X^[^^21^^,^^53^^]^ (4)
***Anthophora*** ^**N**^			X^[^^21^^]^ (1)
***Chelostoma*** ^**N**^			X^[^^21^^]^ (1)
***Colletes*** ^**N**^			X^[^^21^^]^ (1)
***Halictus*** ^**N**^			X^[^^21^^]^ (1)
***Heriades*** ^**N**^			X^[^^21^^,^^53^^]^ (1)
***Hylaeus*** ^**N**^			X^[^^21^^]^ (1)
***Lasioglossum*** ^**N**^			X^[^^21^^]^ (3)
***Melipona*** ^**N**^			X^[^^72^^]^ (5)
***Melitta*** ^**N**^			X^[^^21^^]^ (1)
***Osmia*** ^**N**^			X^[^^21^^,^^53^^]^ (3)
***Scaptotrigona*** ^**N**^			X^[^^72^^]^ (1)
***Tetragonisca*** ^**N**^			X^[^^72^^]^ (1)

Genera with a single or multiple species with a documented *Nosema* species infection. “D” is for domesticated species, and “N” is for native. The number of bee species with a documented infection are in parenthesis, those with “>” are from studies where multiple species of the genus were found infected but not identified below the generic level (see S1 Table for an expanded list of species and geographic distribution). Study citations are written as superscripts.

## Pathogen spillover

While *Nosema* species are spread within eusocial colonies via the fecal–oral pathway, this also leads to the spread of the pathogen through floral resource contamination. When an infected bee visits a floral resource and defecates, the resource is now contaminated and can lead to what is called pathogen spillover and is defined as the transmission of diseases from domesticated animals to wildlife living in close proximity [25]. Any bees that subsequently visit the resource, native or domesticated, are now at risk for infection. This can lead to infection of the host, which will thereby spread the pathogen to other floral resources, which puts the bee community at risk of not only infection and potential fitness consequences but can spread the pathogen throughout the foraging range of the infected bee with a compounding effect. As this spread can lead to a broad landscape pathogen load, there is the potential for significantly reduced pollinator efficacy. Additionally, pathogen spillover can lead to extinction events of small populations that lack defenses against novel pathogens, reverse spillover back to domesticated animals, and evolution of novel strains [25,26,27].

## Management and future directions

Given the consequences of *Nosema* infections, the ability to control the pathogen load within infected bees is of utmost necessity. Historically, the antifungal pesticide Fumagilin-B produced by Medivet Pharmaceuticals Ltd. was the most effective and widespread treatment of *Nosema* infections within managed hives. However, in 2018, Medivet Pharmaceuticals Ltd. announced that due to the cessation of production of the precursors to Fumagilin-B, the company was ceasing production of the compound. This has led to increased research on alternatives for the management of the disease. While breeding for *Nosema* resistant lines of honey bees has been conducted for over a decade with some success [73], chemical alternatives are also being investigated. One such investigation [74] showed that the combination of aqueous extracts of *Artemisia dubia* (Asteraceae, Plantae) and *Aster scaber* (Asteraceae, Plantae) worked best at inhibiting *N*. *ceranae* spore proliferation. Continued exploration and testing of Anti-*Nosema* compounds is necessary, as management of these fungi will most likely require a combination of solutions. A recent review by Burnham [75] efficiently summarized the breadth of treatments being investigated that includes small molecules, RNA interference, extracts and supplements, and microbial supplements. In addition to continued research into treatments for *Nosema* diseases, further environmental surveys must be conducted to determine the distribution of *Nosema* species in both managed and wild bees. Particular focus should be given to the investigation of the pathogen’s distribution and impact on wild, native bees, although this is logistically difficult. Through better understanding of the impact and distribution of these pathogens on native bee communities, better management strategies for domesticated and native bees and the ecosystems they serve will be of vital importance.

## Supporting information

S1 Table *Nosema* species infecting species of bees. Species of *Nosema* infecting different bee species with the location and corresponding citation.
(XLSX)
